# Gaseous Air Pollutants and Hospitalization for Respiratory Disease in the Neonatal Period

**DOI:** 10.1289/ehp.9044

**Published:** 2006-08-03

**Authors:** Robert E. Dales, Sabit Cakmak, Marc Smith Doiron

**Affiliations:** 1 University of Ottawa, Ottawa, Ontario, Canada; 2 Ottawa Hospital Research Institute, Ottawa, Ontario, Canada; 3 Health Canada, Ottawa, Ontario, Canada

**Keywords:** carbon monoxide, hospital admission, infants, ozone, sulfur dioxide

## Abstract

**Objective:**

Current levels of ambient air pollution are associated with morbidity and mortality in the general population. To determine the influence of gaseous air pollutants on neonatal respiratory morbidity, we tested the association between daily respiratory hospitalizations and daily concentrations of ambient air pollution gases: ozone, carbon monoxide, sulfur dioxide, and nitrogen dioxide, in 11 large Canadian cities.

**Study Design:**

Daily time-series analyses were employed and results were adjusted for day of the week, temperature, barometric pressure, and relative humidity.

**Results:**

The percent increases in hospitalization associated with an increase in air pollution equivalent to its interquartile range were 3.35 [95% confidence interval (CI), 1.73–4.77] for O_3_, 2.85 (95% CI, 1.68–4.02) for NO_2_, 1.66 (95% CI, 0.63–2.69) for SO_2_, and 1.75 (95% CI, 0.48–3.02) for CO. The independent effect of all pollutants combined was 9.61% (95% CI, 4.52–14.7%).

**Conclusion:**

Our results suggest that neonates are experiencing adverse effects of air pollution at current levels in Canada, and that accounts for a significant proportion of hospitalizations in this subgroup.

Current levels of ambient air pollution in the Americas, Europe, and Australia have been associated with increased respiratory and cardiac morbidity and mortality in the general population (Bascom et al. 1996; Dockery and Pope 2002; Morgan et al. 1998). The primary focus has usually been either adults or children of all ages combined, but research is needed to know whether the results are generalizable to more narrowly defined age groups which have special characteristics. Associations between ambient air pollution and pregnancy outcomes, including preterm birth, low birth weight, and intrauterine growth restriction (IUGR), have been studied in several countries (Ritz et al. 2002; Wang et al. 1997). Liu et al. (2003) found that maternal exposure to increased concentration of gaseous air pollutants, sulfur dioxide, nitrogen dioxide, and carbon monoxide during pregnancy were positively associated with preterm birth and IUGR. The number of SIDS (sudden infant death syndrome) deaths increased on days of high gaseous air pollution in Canada (Dales et al. 2004). Neonates, 0–28 days of age, have a unique spectrum of morbidity including clinical presentations such as “acute life-threatening events” and “respiratory distress syndrome.” To understand better the contribution of pollution to neonatal morbidity, we used time-series studies to test the hypothesis that gaseous air pollutants are associated with hospital admissions for respiratory diseases among neonates in 11 large Canadian cities. We compared hospitalization rates on higher and lower air pollution days for the same population. As stated by Peng et al. (2006) the results from multicity time-series studies are robust against alternative methodologies for confounding adjustment. To bias the results, a variable would need to be associated with daily changes in air pollution and also be a risk factor for neonatal hospitalization for asthma, making confounding unlikely.

## Methods

### The study population

The study population was all emergency admissions to hospital for respiratory disease in patients from birth to the age of 27 days between 1 January 1986 and 31 December 2000 in 11 of the largest Canadian cities from the Atlantic to Pacific coasts: Calgary, Edmonton, Halifax, London, Hamilton, Ottawa, Saint John, Toronto, Vancouver, Windsor, and Winnipeg. The hospitalization data were provided by the Canadian Institute for Health Information (http://secure.cihi.ca/cihiweb/splash.html), which collects information on all urgent and emergency admissions in Canada; the availability of this information is made possible by the fact that acute care general medical hospitals in Canada are publicly funded. There were a total of 9,542 hospital discharge records for cases in which the principal reason for hospitalization was a respiratory disorder described by the *International Classification of Disease, 9th Revision* (ICD-9; World Health Organization 1975) code: asphyxia (799.0), respiratory failure (799.1), dyspnea and respiratory abnormalities (786.0), respiratory distress syndrome (769), unspecified birth asphyxia in live-born infant (768.9), other respiratory problems after birth (770.8), and pneumonia (486).

### Meteorologic and air pollution data

For each city, the following daily data were provided by Environment Canada and the National Air Pollution Monitoring System (http://www.mb.ec.gc.ca/index.en.html): mean, maximum, and minimum temperature; mean relative humidity; maximum 24-hr change in barometric pressure; and daily measurements of SO_2_, NO_2_, CO, and ozone throughout the 15-year study period. Most cities had several monitoring stations. Averaging data over all monitors within each city created daily summary pollution exposure measures. Daily average values for NO_2_, SO_2_, CO, and O_3_ were used in our analysis. Twenty-four–hour averaged particulate matter < 10 μm in median aerodynamic diameter (PM_10_) and sulfates (SO_4_) were measured every sixth day.

## Statistical Methods

The air pollution, neonatal respiratory hospital admission, and weather variables, measured over the course of the study, were each expressed as a time series. We correlated daily variations in the number of neonatal hospitalization with daily variations in ambient concentrations of air pollutants by using a random-effects regression model for count data (Burnett et al. 1997). Because the response of interest is counts, the residual variation was assumed to be proportional to the expected response, accommodating overdispersion or underdispersion relative to Poisson variation. Neonatal respiratory admissions, air pollution, and weather can vary seasonally and may have other unexplained time trends that can cause confounding (Peng et al. 2006). We adjusted for temporal trends in neonatal respiratory admissions, including day of the week, and effects of weather on neonatal respiratory admissions using piecewise constant functions that varied by 30, 90, 180, 270, and 365 days throughout the study period. This approach, used to address the low counts of neonatal respiratory admissions in some cities, has been previously used by Dales et al. (2004) to study SIDS.

We then selected a model with time period that either minimized the Akaike Information Criterion (AIC; Akaike 1973), or maximized the evidence that the model residuals did not display any type of structure, including serial correlation, using Bartlett’s test. We examined serial correlation structure in the residuals after fitting models with functions that were constant over time intervals. The above steps were implemented separately for each city.

Using S-PLUS (version 6.2; Insightful, Seattle, WA, USA), we tested the association between weather and respiratory admissions with a nonlinear model and natural spline functions of weather variables to remove unwanted temporal trends. These variables included daily minimum and maximum temperature, mean daily temperature, 24-hr change in barometric pressure, and mean relative humidity. Three lags (0, 1, and 2 days) were examined for each of these weather variables. The model containing the latter three weather variables minimized the AIC and was selected as the predictive weather model for daily neonatal respiratory hospitalization. Finally, air pollutants, with lags, were added to the model containing weather variables. We tested time lags between daily pollution concentration and daily neonatal respiratory admissions of 0–5 days. Results for the lag associated with the largest T ratio—the ratio of percentage increase in admissions to the standard error.

Once the final model was selected, the percent change in daily hospitalization and the accompanying standard errors in relative risks were generated for each city. The results for each city were pooled. We calculated the estimates of the pooled effect size mean ± SE by using a fixed- or random-effects model. This weights the individual city effects by the inverse of the between and within-city variance. We used a random-effects model whenever the between-city variance was > 0, even if the heterogeneity among effect-size estimates for individual cities was not statistically significant. We assessed the presence of statistical heterogeneity in effect size between cities using the chi-square statistic. Pooled estimates were expressed as the percentage increase in neonatal hospitalization associated with an increase in the concentration of air pollutants equivalent to the interquartile range among all cities (Burnett et al. 1995). As stated by Peng et al. (2006), the results from multicity time-series studies are robust against alternative methodologies for confounding adjustment. To bias the results, a variable would need to be associated with daily changes in air pollution and also be a risk factor for neonatal hospitalization for asthma, making confounding unlikely.

## Results

The frequency of occurrence of the ICD codes used in this study, presented in Table 1, was relatively low over the 15-year period of observation, with respiratory distress syndrome being the most common of the respiratory diagnoses studied and asphyxia the least common.

Details on population size and the mean of air pollution and meteorologic data are presented in Table 2. O_3_ concentrations ranged from 13.3 ppb in Vancouver to 23.1 ppb in Saint John, with a population-weighted average of 17.0. NO_2_ concentrations ranged from 9.2 ppb in Saint John to 25.6 ppb in Calgary, with a pooled average of 21.8. SO_2_ concentrations ranged from 1.2 ppb in Winnipeg to 10.1 in Halifax, with a pooled average of 4.3 ppb, and CO concentrations ranged from 0.4 in London and to 1.2 ppm in Toronto, with a pooled average of 1.0 ppm.

Pairwise Pearson correlations between pollutants were quite variable between cities. Correlations between NO_2_, SO_2_, and CO were all positive although they varied from 0.13 to 0.76 (Table 3). Correlations between PM_10_ and O_3_ and other pollutants varied from negative to positive, −0.55 to 0.71.

We tested the association between air pollutants and hospitalization using lags of 0–5 days. Averaged over cities, the mean lags providing the greatest association were 2 days for O_3_, 2 days for SO_2_, 1 day for NO_2_, and 1.8 days for CO. Based on minimization of the AIC, temperature was included in the models for Edmonton and Halifax. Relative humidity on the same day was included in all models except the two aforementioned cities. In addition, relative humidity lagged 1 day was included for Calgary, Vancouver, Windsor, and Winnipeg. Change in barometric pressure over the day did not enter the models.

All individual pollutants were associated with increased respiratory hospitalizations, with NO_2_ having the strongest effect whether or not adjusted for the other gases (Table 4). The independent effects of each gas remained stable whether or not adjusted for the effects of the other gases. The percent increases in hospitalization associated with an increase in air pollution equivalent to its interquartile range were 3.35 [95% confidence interval (CI), 1.73–4.77] for O_3_, 2.85 (95% CI, 1.68–4.02) for NO_2_, 1.66 (95% CI, 0.63–2.69) for SO_2_, and 1.75 (95% CI, 0.48–3.02) for CO. The total effect of all gaseous pollutants was a 9.61% (4.52–14.7%) increase in respiratory admissions, with an increase in pollutants equivalent to their interquartile range.

To determine the degree to which PM_10_ may have confounded the findings, we restriced the analyses to every sixth day, when these pollutants were measured during the years 1986–2000. The gases remained statistically significant, with the largest difference in effect-size estimate found for the association between hospitalizations and SO_2_: 2.06% (95% CI, 1.04–3.08%) for the daily model versus 1.41% (95% CI, 0.35–2.47%) for the model restricted to every sixth day.

## Discussion

Although admissions for respiratory disease are uncommon in neonates compared with adults, we were able to detect a significant association with gaseous air pollutants. If the association was causal, air pollution at ambient levels seen in Canada may account for 16% of admissions. Our results are consistent with previously observed associations in older children and adults between air pollution and respiratory symptoms, lung function, hospitalization, and mortality. The two strongest effects observed were with NO_2_ and O_3_.

A large meta-analysis of studies from around the world found consistent associations between mortality and NO_2_ even when adjusted for other pollutants (Stieb et al. 2002). Associations have also been found linking all air pollutant gases with lung function impairment in both children and adults (Gauderman et al. 2000; Peters et al. 1999a, 1999b). In other observational studies, acute changes in NO_2_ have been associated with sudden infant death syndrome in Canada, infant mortality in Mexico City, and asthma symptoms in children (Dales et al. 2004; Delfino et al. 2002; Loomis et al. 1999). Exposures during pregnancy have been associated with low birth weight in Seoul, Korea, and IUGR in Vancouver, Canada (Ha et al. 2001; Liu et al. 2003). Controlled human exposures to NO_2_ have demonstrated small increases in bronchial reactivity (Folinsbee 1992) and an enhanced effect of allergen-induced bronchoconstriction (Strand et al. 1998). In mice, NO_2_ exposure increases morbidity from *Mycoplasma pulmonis* (Parker et al. 1989) and mortality from *Klebsiella Pneumoniae* (Ehrlich 1966; Gauderman et al. 2000; Peters et al. 1999a, 1999b; Stieb et al. 2002). The effects of NO_2_ in large observational studies have been found at lower concentrations than those investigated in laboratory studies. Several possible reasons exist. The much larger sample size in population studies allows the ability to detect smaller effects. The laboratory studies control for other confounding variables better than can be done in an observational study. Perhaps NO_2_ is simply a marker of other toxicants in a complex mixture of pollutants that share a common source. Nevertheless, our findings of increased neonatal morbidity from air pollution gases are consistent with other studies of early life.

Particulates such as NO_2_ are also markers of exposure to vehicular traffic. Although our study focused on air pollution gases, we had an opportunity to assess the effects of PM_10_ measured every sixth day. A 10-μg/m^3^ increase in PM_10_ has been associated with a 0.51–1% increase in all-cause mortality (Burnett et al. 1995) and a 1–4% increase in hospital visits (Pope et al. 1995). Our results were of similar magnitude, although in a different age group. We found a 1.5–2.1% increase in hospitalization among neonates associated with a 15-μg/m^3^ increase in PM_10_, of similar magnitude to studies of other age groups. A more recent cohort study of cystic fibrosis patients found that living in an area with a 10-μg/m^3^ higher concentration of PM_10_ was associated with an 8% (95% CI, 2–15%) increase in the odds of at least two disease exacerbations requiring hospitalization or home intravenous antibiotics (Goss et al. 2004).

For a 100-ppb increase in the daily one-hour maximum of O_3_, Thurston and Ito (1999) reported that the relative risk of respiratory hospitalization for all ages was 1.18 (95% CI, 1.1–1.3), and the 1-sec forced expiratory volume in children attending summer camps decreased by 50.0 (SE = 7.0) mL.

Most of the literature on air pollution and health includes both adults and children together. The many reports of air pollution and children’s health either excluded neonates (Farrow et al. 1997; Gouveia and Fletcher 2000; Ha et al. 2003; Woodruff et al. 1997) or did not study them separately from all children (Burnett et al. 2001, 1994; Loomis et al. 1999). Yet neonates have a unique set of respiratory diagnoses such as acute life-threatening events and respiratory distress syndrome, whereas older children commonly suffer from asthma, croup, and bronchiolitis, and adults are admitted with asthma, pneumonia and chronic obstructive pulmonary disease. We could not find previous studies of air pollution and hospitalization for neonatal respiratory disease, but the findings are consistent with the few published studies focusing on neonatal mortality. Lin et al. (2004) reported that a 23.3-μg/m^3^ increase in PM_10_ was associated with a 4% (95% CI, 2–6%) increase in total daily mortality in neonates in São Paulo, Brazil, whereas a 9.2-μg/m^3^ increase in SO_2_ was associated with a 6% (95% CI, 4–8%) increase in mortality. The combined effects of the two pollutants was 6.3% (95% CI, 6.1–6.5%), indicating that the SO_2_ alone could explain most of the effect. No significant effects, and weather variables. effect of NO_2_, CO, or O_3_ could be detected, although an average of 6.1 deaths occurred daily over the 1,096 days of study.

Bobak and Leon (1999) performed a case–control study of live births in the Czech Republic between 1989 and 1991. Each infant death was matched with 20 living infants who were of the same sex and born on the same day. No significant effect of total suspended particulates (TSP), SO_2_, or NO_2_ was found in the 1,684 cases of death in the neonatal period, adjusted for unspecified socioeconomic factors, gestational age, and birth weight. Exposure to environmental tobacco smoke was not known. TSP and SO_2_ were significantly associated with respiratory (*n* = 111 cases) but not all-cause deaths (*n* = 810) in the postneonatal period, with rate ratios of 1.95 (95% CI, 1.1–3.5) and 1.74 (95% CI, 1.0–3.0) respectively for an increase of 50 μg/m^3^.

Lipfert et al. (2000) matched the U.S. Environmental Protective Agency’s air pollution database with the National Linked Birth/Infant Death Data Set of the National Center for Health Statistics for 1990. Annual averages of air pollution were estimated for each infant based on the county of birth. SO_4_ and PM_10_ were positively associated with total neonatal deaths, and PM_10_ was associated with neonatal respiratory deaths, adjusted for mother’s smoking, marital status, race, infant’s month of birth, and average heating degree days. There were no associations with CO, and associations with SO_2_ and NO_2_ were not reported.

Dales et al. (2004) reported that between 1984 and 1999, daily increases in SO_2_ and NO_2_ equivalent to their interquartile ranges were associated with an 18% increase in daily SIDS. The study was based on infant deaths occurring in the same Canadian cities on which the present study is based.

Even earlier in life, there is emerging evidence suggesting adverse effects of air pollution. In British Columbia, lower birth weight has been found in areas of higher air pollution adjusting for maternal age**,** parity, infant sex, gestational age or birth weight, and month of birth (Liu et al. 2003). However, socioeconomic status, a potential confounder, was not accounted for in the study. In conclusion, current Canadian levels of ambient air pollution are associated with a significant proportion of hospitalizations for respiratory disorders in neonates. This is consistent with findings of adverse effects in both older and younger age groups.

## Figures and Tables

**Table 1:** Frequency of occurrence of respiratory diagnoses in 11 Canadian cities, 1 January 1986 to 31 December 2000.

City	1996 Census population (× 10^5^)	Asphyxia	Respiratory failure	Dyspnea and respiratory abnormalities	Respiratory distress syndrome	Unspecified birth asphyxia in live born	Other respiratory problems after birth	Pneumonia	All respiratory diagnoses
Calgary	7.68	2	13	209	272	19	270	26	811
Edmonton	6.16	0	9	215	143	53	268	54	742
Halifax	1.80	0	1	46	1	0	14	8	70
Hamilton	2.45	1	6	181	107	4	76	27	402
London	1.25	1	11	82	21	4	110	24	253
Ottawa	9.39	1	14	173	99	26	104	32	449
Saint John	1.02	0	1	57	1	0	12	3	74
Toronto	23.85	9	22	821	789	189	543	212	2,585
Vancouver	18.32	4	30	555	1,510	82	367	111	2,659
Windsor	1.98	2	4	105	22	3	34	21	191
Winnipeg	6.88	0	15	97	21	11	110	96	350
Total		20	126	2,541	2,986	391	1,908	614	8,586

Census data from Statistics Canada 1996.

**Table 2:** Population size, 24-hr mean air pollution levels (5th, 95th percentiles), and weather variables for 11 Canadian cities, 1 January 1986 to 31 December 2000.

City	O_3_ (ppb)	NO_2_ (ppb)	SO_2_ (ppb)	CO (ppb)	Mean temperature (°C)	24-hr change in barometric pressure	% Relative humidity
Calgary	17.8 (4.7, 32.3)	25.6 (13.3, 41.0)	3.6 (1.0, 8.0)	0.9 (0.4, 2.0)	4.5 (−15.5, 18.4)	0.0 (−1.1, 1.13)	61.2 (37, 86)
Edmonton	17.0 (4.0, 33.1)	24.6 (11.5, 43)	2.7 (0, 6.0)	1.1 (0.4, 2.4)	3.0 (−19.5, 18.1)	0.0 (−1.2, 1.2)	68.6 (47, 88)
Halifax	20.8 (9, 35)	15.1 (3, 28)	10.1 (2, 23)	0.8 (0.3, 1.7)	6.4 (−10.4, 10.3)	0.0 (−1.7, 1.6)	77.5 (54, 96)
Hamilton	19.0 (3.3, 41.8)	20.8 (11, 34)	8.2 (1.7, 17.5)	0.9 (0.2, 1.6)	7.9 (−9.4, 22.8)	0.0 (−1.3, 1.3)	73.5 (50, 95)
London	22.3 (6, 46)	20.0 (8, 35)	3.7 (0, 11)	0.4 (0, 1.2)	7.9 (−9.7, 22.9)	0.0 (−1.25, 1.27)	75.7 (55, 93)
Ottawa	16.4 (4.5, 31.0)	21.2 (7, 38)	3.9 (0, 10)	0.9 (0.2, 1.9)	6.3 (−15, 23)	0.0 (−1.5, 1.5)	69.4 (46, 91)
Saint John	23.1 (10.7, 38.5)	9.2 (2, 21)	8.3 (0.5, 23.5)	0.7 (0.1, 1.7)	5.1 (−12.6, 18.6)	0.0 (−1.6, 1.5)	75.4 (52, 95)
Toronto	18.3 (5, 36.7)	25.1 (14, 39)	4.5 (0.2, 11.3)	1.2 (0.6, 1.9)	8.1 (−9.6, 23.4)	0.0 (−1.4, 1.3)	71.9 (52, 90)
Vancouver	13.3 (3.2, 24.9)	19.0 (11.4, 30.2)	4.6 (1.2, 9.8)	0.9 (6.4, 1.9)	10.5 (1.5, 19.1)	0.0 (−1.1, 1.2)	79.3 (64, 94)
Windsor	18.7 (3, 42)	24.9 (11, 41)	7.6 (1.7, 15.7)	0.8 (0, 1.5)	9.8 (−7.3, 25.1)	0.0 (−1.2, 1.3)	70.8 (51, 91)
Winnipeg	18.5 (6, 34)	15.2 (6, 28)	1.2 (0, 3.5)	0.6 (0.3, 1.0)	3.1 (−22.6, 22.1)	0.0 (−1.4, 1.4)	71.9 (49, 91)
Population weighted average	17.0	21.8	4.3	1.0	7.2	48.1	72.3

**Table 3:** Range of Pearson pairwise correlations between pollutants by city for 11 Canadian cities, 1 January 1986 to 31 December 2000.

	PM_10_	O_3_	NO_2_	SO_2_
PM_10_
O_3_	−0.29 to 0.41			
NO_2_	−0.26 to 0.69	−0.55 to 0.05		
SO_2_	−0.09 to 0.61	−0.41 to 0.13	0.20 to 0.67	
CO	−0.13 to 0.71	−0.54 to −0.01	0.13 to 0.76	0.19 to 0.66

**Table 4:** The pooled estimate of percent increase (95% CI) in neonatal respiratory hospital admissions associated with an increase in air pollution concentration increase equal in magnitude to its interquartile range. Data from 11 Canadian cities, 1 January 1986 to 31 December 2000.

Air pollutants	Interquartile range	Single-pollutant model^a^	Multi-pollutant model^b^	Multipollutant model^b^ restricted to days with PM_10_ measures
O_3_ (ppb)	12.0	3.21 (1.72 to 4.70)	3.35 (1.73 to 4.77)	2.67 (0.98 to 4.39)
NO_2_ (ppb)	10.0	2.94 (1.93 to 3.95)	2.85 (1.68 to 4.02)	2.48 (1.18 to 3.80)
SO_2_ (ppb)	3.8	2.06 (1.04 to 3.08)	1.66 (0.63 to 2.69)	1.41 (0.35 to 2.47)
CO (ppm)	0.5	1.75 (0.63 to 2.87)	1.75 (0.48 to 3.02)	1.30 (0.13 to 2.49)
PM_10_ (μg/m^3^)	15.2	2.13 (−0.5 to 4.76)	1.45 (−1.90 to 4.80)	

a Effect size is adjusted for long-term temporal trends, day of week effects, and weather variables.

b Effect size is adjusted for other gases and size is adjusted for long-term temporal trends, day of week effects, and weather variables.
